# The Biological Observation Matrix (BIOM) format or: how I learned to stop worrying and love the ome-ome

**DOI:** 10.1186/2047-217X-1-7

**Published:** 2012-07-12

**Authors:** Daniel McDonald, Jose C Clemente, Justin Kuczynski, Jai Ram Rideout, Jesse Stombaugh, Doug Wendel, Andreas Wilke, Susan Huse, John Hufnagle, Folker Meyer, Rob Knight, J Gregory Caporaso

**Affiliations:** 1 Biofrontiers Institute, University of Colorado, Boulder, CO, USA; 2 Department of Chemistry & Biochemistry, University of Colorado, Boulder, CO, USA; 3 Second Genome, San Bruno, CA, USA; 4 Department of Computer Science, Northern Arizona University, Flagstaff, AZ, USA; 5 Argonne National Laboratory, Argonne, IL, USA; 6 Marine Biological Laboratory, Woods Hole, MA, USA; 7 Howard Hughes Medical Institute, Boulder, CO, USA

**Keywords:** Microbial ecology, Comparative genomics, Metagenomics, QIIME, MG-RAST, VAMPS, BIOM

## Abstract

**Background:**

We present the Biological Observation Matrix (BIOM, pronounced “biome”) format: a JSON-based file format for representing arbitrary observation by sample contingency tables with associated sample and observation metadata. As the number of categories of comparative omics data types (collectively, the “ome-ome”) grows rapidly, a general format to represent and archive this data will facilitate the interoperability of existing bioinformatics tools and future meta-analyses.

**Findings:**

The BIOM file format is supported by an independent open-source software project (the biom-format project), which initially contains Python objects that support the use and manipulation of BIOM data in Python programs, and is intended to be an open development effort where developers can submit implementations of these objects in other programming languages.

**Conclusions:**

The BIOM file format and the biom-format project are steps toward reducing the “bioinformatics bottleneck” that is currently being experienced in diverse areas of biological sciences, and will help us move toward the next phase of comparative omics where basic science is translated into clinical and environmental applications. The BIOM file format is currently recognized as an Earth Microbiome Project Standard, and as a Candidate Standard by the Genomic Standards Consortium.

## Background

Advances in DNA sequencing have led to exponential increases in the quantity of data available for “comparative omics” analyses, including metagenomics (e.g., [1,2]), comparative genomics (e.g., [3]), metatranscriptomics (e.g., [4,5]), and marker-gene-based community surveys (e.g., [6,7]). With the introduction of a new generation of "benchtop sequencers" [8], accessible to small research, clinical, and educational laboratories, sequence-based comparative omic studies will continue to increase in scale. The rate-limiting step in many areas of comparative omics is no longer obtaining data, but analyzing that data (the “bioinformatics bottleneck”) [9,10]. One mechanism that will help reduce this “bioinformatics bottleneck” is standardization of common file formats to facilitate sharing and archiving of data [11].

**Figure 1:**
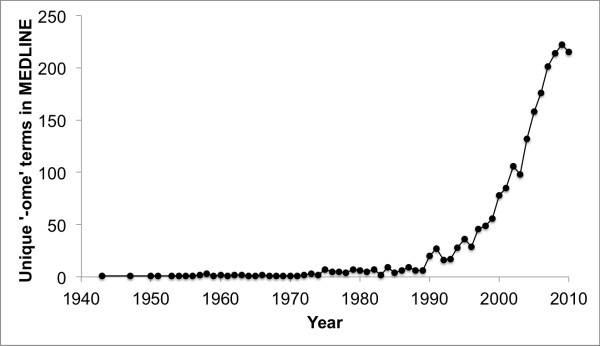
**Growth of the “ome-ome”, or the types of “omic” data, over time based on mentions in Medline abstracts.** Chao1 analysis indicates that there may be over 3,000 “omes”: however, given the well-known limitations of such non-parametric extrapolation techniques, we can only wonder how many “omes” remain to be discovered as technological advances usher in a new era of “ome-omics”.

As with the increasing prevalence of high-throughput technologies in the biological sciences, the categories of comparative omics data, which we collectively term the “ome-ome”, are rapidly increasing in number (Figure 1). Researchers are relying on more types of omics data to investigate biological systems, and the coming years will bring increased integration of different types of comparative omics data [2,12]. A common data format will facilitate the sharing and publication of comparative omics data and associated metadata and improve the interoperability of comparative omics software. Further, it will enable rapid advances in omics fields by allowing researchers to focus on data analysis instead of on formatting data for transfer between different software packages or reimplementing existing analysis workflows to support their specific data types.

Despite the different types of data involved in the various comparative omics techniques (e.g., metabolomics, proteomics, or microarray-based transcriptome analyses), they all share an underlying, core data type: the “sample by observation contingency table”, or the matrix of abundances of observations on a per-sample basis. In marker gene surveys, this table contains counts of OTUs (Operational Taxonomic Units) or taxa on a per-sample basis; in metagenome analyses, counts of orthologous groups of genes, taxa, or enzymatic activities on a per-metagenome basis; in comparative genomics, counts of genes or orthologous groups on a per-genome basis; and in metabolomics, counts of metabolites on a per-sample basis. Many tools have been developed to analyze these contingency tables, but they are generally focused on a specific type of study (e.g., QIIME for marker gene analysis [13], MG-RAST for metagenome analysis [14], VAMPS for taxonomic analysis [15]). However, many techniques are applicable across data types, for example rarefaction analyses (i.e., collector curves). These are frequently applied in microbiome studies to compare how the rate of incorporation of additional sequence observations affects the rate at which new OTUs are observed. This allows us to determine whether an environment is approaching the point of being fully sampled (e.g., [13]). Rarefaction curves could similarly be applied in comparative genomics to study the rate of discovery of new gene families, as done in [16]; a researcher could compile a contingency table of genomes (samples) by genes (observations) and use a rarefaction curve to determine how quickly new gene families were accumulating as new genome sequences are added. A standard format for biological sample by observation contingency tables will support the use of bioinformatics pipelines for different data types than those they were initially designed for (e.g., QIIME could be applied to generate rarefaction curves for proteomic data, or MG-RAST could output metatranscriptome tables). Adoption of this standard will additionally facilitate the adoption of future analysis pipelines, as users can then directly apply those pipelines to their existing data.

In many existing software packages (e.g., [13,14]), contingency tables are represented as tab-separated text, but minor syntactic differences prevent easy exchange of data between tools. For example, differing representation of samples and observations as either rows or columns, and the mechanism for incorporating sample or observation metadata (if possible at all), cause the formats used by different software packages to be incompatible. Additionally, in many of these applications a majority of the values (frequently greater than 90 %) in the contingency table are zero, which is taken to mean that the corresponding “observation” was not observed in the corresponding sample. The fraction of the table that has non-zero values is defined as the "density", and thus a matrix with a low number of non-zero values is said to have a low density. As data sets continue to increase in size, “dense” representations of these tables, where all values are represented (in contrast to “sparse” representations, where only non-zero values are represented), result in an increasingly inefficient use of disk space. For example, marker gene survey OTU tables with many samples (such as the one presented in Additional file 1: Table S1 containing 6,164 samples and 7,082 OTUs) can have as few as 1 % non-zero values. As the collection of samples becomes more diverse, these tables become even sparser and their size (both on disk and in memory) becomes a considerable barrier to performing meta-analyses.

Sample and observation metadata are essential for the interpretation of omics data, and for facilitating future meta-analyses. Two projects have recently arisen to address the need for metadata standards: MIxS [17], which defines what metadata should be stored for diverse sequence types, and ISA-TAB [11], which defines a file format for storing that metadata. A standard file format for representing sample by observation contingency tables could compliment these existing standards by providing a means for associating MIxS-compliant metadata provided in ISA-TAB format with samples and observations.

The Biological Observation Matrix (BIOM, pronounced “biome”) file format has been developed with input from the QIIME, MG-RAST, and VAMPS development groups. The BIOM file format is based on JSON [18], an open standard for data exchange. The primary objectives of the BIOM file format are presented in Additional file 2. In addition to consolidating data and metadata in a single, standard file format, the BIOM file format supports sparse and dense matrix representations to efficiently store these data on disk. The OTU table with 6,164 samples and 7,082 OTUs mentioned above contains approximately 1 % non-zero values. Because zero-values are not included in the sparse BIOM-formatted file, representing the same information in this format requires 14 times less space than with a tab-separated text file (Supplementary File 1). As a sparse matrix increases in size or decreases in density (e.g., in an Illumina sequencing run versus a 454 sequencing run), this difference in file size will further increase.

To support the use of the BIOM file format, the format specifications and an open-source software package, biom-format, are available at http://biom-format.org. Included with the format specification is a format validator, and included in the software package is a script to easily convert BIOM files to tab-separated text representations (which can be useful when working with spreadsheet programs) and Python objects to support working with this data. Additional file 3 presents a comparison of QIIME software for processing a contingency matrix as a 2D array (derived from QIIME 1.4.0) versus using the biom-format objects (derived from QIIME 1.4.0-dev). The biom-format software package will additionally serve as a repository where other developers can submit implementations of these objects in other languages.

## Data description

To compare the relative size of storing sample by observation contingency tables in sparse BIOM-formatted files versus tab-separated files, we extracted 60 QIIME OTU tables from the QIIME database. Each observation (OTU) in these tables contains a single metadata entry corresponding to the taxonomy assigned to the OTU, and the tab-separated files were formatted in “Classic QIIME OTU table” format (i.e., the format generated by QIIME 1.4.0 and earlier). Example files in both BIOM format and classic QIIME OTU table format are available in Additional file 4: Data 1.

## Analyses

**Figure 2:**
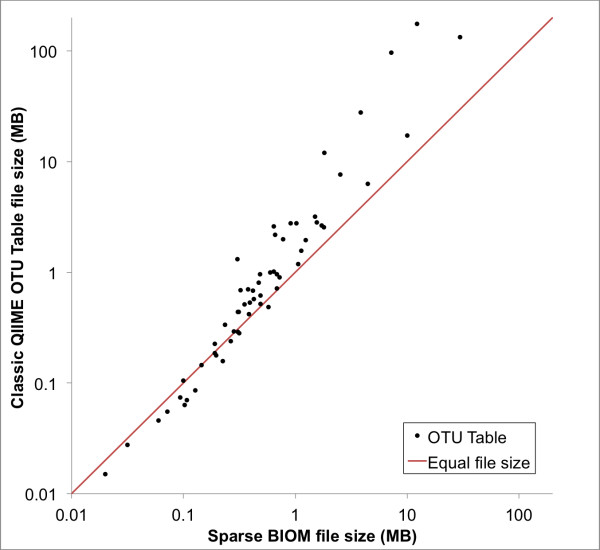
**Size of sparse BIOM formatted file versus size of QIIME “classic” OTU Table formatted file, for 60 independent microbiome studies currently stored in the QIIME database at** **http://www.microbio.me/qiime**

The OTU tables selected for this study ranged in size from 6 samples by 478 OTUs (BIOM size: 0.10 MB; classic QIIME OTU table size: 0.06 MB) up to 6,164 samples by 7,082 OTUs (BIOM size: 12.24 MB; classic QIIME OTU table size: 175.76 MB). In the latter case, at approximately 1 % density there are 100-fold fewer counts in the sparse OTU table, but the file size is only 10-fold (rather than 100-fold) smaller for BIOM-formatted versus tab-separated text. This discrepancy arises because the matrix positions must be stored with the counts in the sparse representation (as *row number, column number, value*; see Additional file 5) but are implied in tab-separated text. The file compression ratio (tab-separated text file size divided by BIOM file size) that is achieved when representing contingency tables in sparse versus dense formats is therefore a function of the density of the contingency table. In the data presented in Figure 2, the density ranges from 1.3 % non-zero values to 49.8 % non-zero values, with a median of 11.1 %. The file compression ratio increases with decreasing contingency table density for this data set (compression ratio = 0.2 × density^-0.8^; R^2^ = 0.9; Additional file 6 Figure S1).

At small file sizes, tab-separated text files represent OTU tables more efficiently than BIOM-formatted files, but starting at approximately 0.2 MB the sparse BIOM representation becomes more efficient (Figure 2). This extra overhead incurred with the sparse representation is negligible (on the order of kilobytes) in cases where the dense representation is more efficient. As contingency table density increases, as may be the case with certain types of comparative omics data, users can format their files in dense BIOM format to avoid inefficiencies with sparse representations. We find that dense representations become more efficient than sparse representations at a density of around 15 % (Additional file 6Figure S1, Additional file 1: Table S1).

In general, a simple tab-separated format will be slightly more efficient for storage than the dense BIOM file format, but will not provide a standard way to store sample and observation metadata or provide interoperability across comparative omics software packages; thus, the BIOM file format will still be advantageous. Similarly, compressing tab-separated text files representing sample by observation contingency tables (e.g., with gzip) can result in a similar degree of compression as converting a dense matrix representation to a sparse representation, but would not provide the additional benefits of the BIOM file format.

## Discussion

The biom-format software package has been designed with three main objectives: to be a central repository for objects that support BIOM-formatted data in different programming languages, to have minimal external dependencies, and to provide an efficient means for representing biological contingency tables in memory along with convenient functionality for operating on those tables. At present we provide Python 2 (2.6 or greater) objects in both dense and sparse representations to allow for efficient storage across a range of densities of the underlying contingency table data. Our goal is to make the biom-format project an open development effort so that other groups can provide objects implemented in different programming languages (ideally with APIs as similar as possible to the Python API).

Managing a community development effort is a challenge. To address this, we will maintain a code repository on GitHub [19] which is currently used for managing many successful collaborative software projects such as IPython, homebrew, and rails. The core BIOM development group will review new additions (in the form of pull requests) and, when they are fully documented and tested, will merge them into the biom-format repository.

A challenge in achieving community adoption of a new standard is convincing users and developers to overcome the learning curve associated with it. To address this, we have fully documented the BIOM file format standard, as well as the motivations for it, on the BIOM format website (http://biom-format.org). The biom-format software project contains a conversion script that allows users to easily move between BIOM-formatted files and tab-separated text files. This allows users to interact with their data in ways they traditionally have (e.g., in a spreadsheet program). To reduce the barrier-to-entry for using the biom-format software, the Python objects in the biom-format package are designed to be easily installable on any system running Python 2.6 or 2.7. To achieve this, biom-format relies only on the Python Standard Library and NumPy (a common dependency for scientific Python applications which is installed by default on Mac OS X and many versions of Linux).

The introduction and refinement of high-throughput sequencing technology is causing a large increase in both the number of samples and the number of observations involved in comparative omic studies (e.g., [6,20]), and sparse contingency tables are therefore becoming central data types in these studies. For example, it is not uncommon to find hundreds of thousands of OTUs in modern microbial ecology studies (unpublished observation based on preliminary analysis of the initial Earth Microbiome Project [20] dataset). Whether these observations represent new biological findings or sequencing error is a contested topic [21-23], but certain poorly characterized environments are hypothesized to contain large reservoirs of yet unknown OTUs [24]. We expect both the number of samples and the number of observations involved in comparative omic studies to continue to grow over the coming years, and an efficient representation of this data that can be easily interrogated across different bioinformatics pipelines will be essential to reducing the bioinformatics bottleneck. Similarly, integrating metadata in BIOM formatted files, ideally based on standards such as MIxS and ISA-TAB, will facilitate meta-analysis across different data types.

The number of categories of comparative omic data (e.g., genomic, metabolomic, pharmacogenomic, metagenomic) is increasing rapidly, and the need to develop software tools specific to each of these data types contributes to the bioinformatics bottleneck. The BIOM file format provides a standard representation of the “sample by observation contingency table”, a central data type in broad areas of comparative omics, providing the means to generally apply tools initially designed for analysis of specific “omes” to diverse “omic” data types. The BIOM file format is currently recognized as an Earth Microbiome Project Standard and a Candidate Standard by the Genomics Standards Consortium, and is being adopted by groups developing comparative omics analysis software. We can embrace the proliferation of omics techniques by using standards such as the BIOM file format to reduce the gap in availability of bioinformatics tools for new domains of omics research. Taken together, these advances are an additional step toward the next phase of comparative omics analysis, in which fundamental scientific findings will increasingly be translated into clinical or environmental applications.

## Methods

### Growth of the ome-ome

In order to evaluate the growth of the “ome-ome” over time we searched a local installation of MEDLINE abstracts (through 2010) and tabulated the number of distinct terms ending in “ome” or “omes” on an annual basis. A list of false positive terms was compiled from the Mac OS ×  10.7.4 built-in dictionary, and an initial pass over MEDLINE to identify irrelevant terms ending in *ome* that are not part of the standard English lexicon (e.g., “trifluorome”, “cytochrome”, “ribosome”). While some false positives are still present, the number of unique “ome” terms being referenced in the biomedical literature is growing rapidly.

### BIOM file format

The BIOM file format version 1.0.0 is based on JSON, an open standard for data exchange for which native parsers in several programming languages are available. JSON was chosen as the basis for the BIOM format as it is a widely accepted and lightweight transmission format used on the Internet since 1999. It is directly translatable into XML if necessary, but embodies less complexity and overhead (in terms of the amount of supporting information that must be included in a valid file).

Several representative BIOM-formatted files and classic QIIME OTU table files used in the analysis presented in Figure 2, Additional file 1: Table S1, and Additional file 6: Figure S1 are provided in a zip file as Additional file 4: Data 1. A full definition of the BIOM format is available at http://biom-format.org.

The BIOM project consists of two independent components. The first component is the BIOM file format specification, which is versioned and available at http://biom-format.org. A BIOM validator script is additionally packaged with the format specification, and allows users to determine if their files are in valid BIOM format. The second component of the BIOM format project is the biom-format software package, which contains general-purpose tools for interacting with BIOM formatted files (e.g., the convert_biom.py script, which allows for conversion between sparse and dense BIOM-formatted files, and for conversion between BIOM-formatted files and tab-separated text files), an implementation of support objects for BIOM data in Python, and unit tests for all software. We hope that the development of similar support objects in other programming languages will become a community effort, which we will manage using the GitHub environment.

## Availability of software

The biom-format project is hosted on GitHub and available at http://www.biom-format.org. The project page can be found at http://github.com/biom-format. biom-format is platform independent, and requires Python 2.6 or 2.7. It is available under GPL v3, and is free for all use. Version 1.0.0 of the biom-format project is available as Supplementary File 2, and available for download on the project page at: https://github.com/downloads/biom-format/biom-format/biom-format-1.0.0.tgz.

## Note from the Editors

A related discussion by Jonathan Eisen on the issues surrounding this work is published alongside this article [25].

## Abbreviations

BIOM: Biological Observation Matrix; QIIME: Quantitative Insights Into Microbial Ecology; MG-RAST: Metagenomic Rapid Annotation using Subsystem Technology; VAMPS: Visualization and Analysis of Microbial Population Structures; OTU: Operational Taxonomic Unit; API: Application Programmer Interface; JSON: JavaScript Object Notation; GPL: GNU Public License.

## Misc

Daniel McDonald and Jose C Clemente contributed equally to this work.

## Competing interests

The authors have declared no competing financial interests.

## Authors’ contributions

All authors designed the BIOM format and provided feedback on the manuscript. DM, JCC, JK, JRR, and JGC developed the biom-format software. DM, JCC, RK, and JGC wrote the manuscript. DM, JCC, RK, and JGC conceived of the project. All authors read and approved the final manuscript.

## Authors’ information

DM, JCC, JK, JRR, JS, DW, RK, and JGC have development and/or leadership roles in QIIME. AW and FM have development and/or leadership roles in MG-RAST. SH and JH have development and/or leadership roles in VAMPS.

## Supplementary Material

Additional file 1: Table S1. OTU table statistics for data included in Figure 2, Additional File 6: Figure S1, and Additional File 7: Data 2. Click here for file

Additional file 2: Initial goals of the biom-format project. Click here for file

Additional file 3: Comparison of QIIME OTU Table collapsing code with native QIIME OTU table data structures (Panels A-D) and biom-format Table objects (Panel E). Panels A-D (combined) provide the same functionality as Panel E. Click here for file

Additional file 4: Data 1. Representative OTU tables in BIOM and classic QIIME OTU table format. Click here for file

Additional file 5: Example BIOM-formatted data. This is an example of a sparse BIOM-formatted OTU table. While type is a required entry in BIOM tables, the BIOM format itself does not change for different data types (e.g., OTU Table, function table, metabolite table). This information is included to allow tools that use BIOM files to determine the data type, if desired. Additional examples are available at: http://biom-format.org/documentation/format_versions/biom-1.0.html#example-biom-files. Click here for file

Additional file 6: Figure S1. Matrix density versions compression ratio. Click here for file

Additional file 7: Data 2. Version 1.0.0 of the biom-format software package. Click here for file
