# Supplementary material for: The Biological Observation Matrix (BIOM) format or: how I learned to stop worrying and love the ome-ome
Source: Gigascience. 2012 Jul 12;1:7. doi: 10.1186/2047-217X-1-7 (PMC3626512; doi:10.1186/2047-217X-1-7)
Supplement: Additional file 1: Table S1. — OTU table statistics for data included in Figure 2, Additional File 6: Figure S1, and Additional File 7: Data 2. [file 2047-217X-1-7-S1.pdf]

Box 1: Goals of the BIOM format.
The initial goals for the BIOM format and `biom-format` software project are as follows:

- The format should be fully generalizable to arbitrary biological sample and observation types, not specific to one or a few data types.
- The contingency table should be representable in either sparse or dense matrix format for file size, load time, and runtime memory considerations. These contingency tables tend to be sparse (i.e., containing mostly counts of zero) for many comparative omics fields.
- Values in the contingency table should be representable either as integers or floating point (i.e., real) numbers to support absolute and relative abundances.
- Sample and observation metadata should be optionally representable. Samples generally have metadata describing environmental parameters (such as 'host-associated' or 'free-living') while observation metadata may describe the taxonomic or functional classification of each observation.
- Information on the data type (e.g., OTU Table, Ortholog Table, Metabolite Table) should be included, and based on terms from a controlled vocabulary. This controlled vocabulary should be easily updatable to support new data types.
- Information on the source of the file should be present in the file including the software package and version that generated the file (e.g., "QIIME version 1.5.0"), and date and time of the file creation.
- The BIOM format should be versioned, and this version information should be included in all BIOM files.
