# Supplementary material for: The Biological Observation Matrix (BIOM) format or: how I learned to stop worrying and love the ome-ome
Source: Gigascience. 2012 Jul 12;1:7. doi: 10.1186/2047-217X-1-7 (PMC3626512; doi:10.1186/2047-217X-1-7)
Supplement: Additional file 2: — Initial goals of the biom-format project. [file 2047-217X-1-7-S2.pdf]

Box 2: Comparison of QIIME OTU Table collapsing code with native QIIME OTU table data structures (Panels A-D) and `biom-format` Table objects (Panel E). Given an OTU table and associated sample metadata, this code collapses sets of samples with the same value for a given metadata entry into a single sample. Here we illustrate the vastly reduced complexity of this operation using `biom-format` Table objects (in QIIME 1.4.0-dev svn revision 2770 and later; Panel E) versus native QIIME objects in QIIME (QIIME 1.4.0 and earlier, Panels A-D). The full version of each example can be found in the QIIME repository using the information in each panel caption.

Panel A: QIIME 1.4.0: `Qiime/scripts/summarize_otu_by_cat.py` (prior to switch to `biom-format` Table objects).

```
    mapping_f = open(mapping_fp,'U')
    otu_table_f = open(otu_table_fp,'U')

    summarized_otu_table = \
     summarize_by_cat(mapping_f,otu_table_f,mapping_category,normalize)

    f = open(output_fp,'w')
    f.write(summarized_otu_table)
    f.close()
```

Panel B: QIIME 1.4.0: `Qiime/qiime/summarize_otu_by_cat.py` (prior to switch to `biom-format` Table objects).

```
def get_sample_cat_info(lines, category):
    cat_by_sample = {}
    sample_by_cat = defaultdict(list)
    meta_dict = {}
    num_samples_by_cat = defaultdict(int)
    label_lists_dict = defaultdict(list)
    mapping_data, header, comments = parse_mapping_file(lines)

    category_labels = header
    index = category_labels.index(category)

    for line in mapping_data:
        categories = line[0:len(category_labels)+1]
        sample = categories[0].strip()
        meta_dict[sample] = [(categories[index],0)]

        cat_by_sample[sample] = [(l.strip(),c.strip()) \
                         for l,c in zip(category_labels,categories)]

        cat_list = []
        for i,(l,c) in enumerate(zip(category_labels,categories)):
            if c not in label_lists_dict[l]:
                label_lists_dict[l].append(c)
            l = l.strip()
            c = c.strip()
            cat_list.append((l,c))
            sample_by_cat[(l,c)].append(sample)
            num_samples_by_cat[(l,c)] += 1

        cat_by_sample[sample] = cat_list

    return cat_by_sample, sample_by_cat, len(category_labels),
meta_dict,label_lists_dict,num_samples_by_cat

```

Panel C: QIIME 1.4.0: `Qiime/qiime/summarize_otu_by_cat.py` (continued; prior to switch to `biom-format` Table objects).

```python
def get_counts_by_cat(lines, num_meta, meta_dict, cat_list,category,num_samples_by_cat,
                      normalize):

    samples_from_mapping = meta_dict.keys()
    norm_otu_table =[]
    sample_counts = defaultdict(int)
    cat_otu_table = []
    otus = []
    taxonomy = []
    sample_ids, otu_ids, otu_table, lineages = parse_otu_table(lines)

    label_list = sample_ids
    if lineages == []:
        is_con = False
    else:
        is_con = True
    for idx, line in enumerate(otu_table):
        new_line = []
        label_dict = defaultdict(int)
        data = line
        to_otu = otu_ids[idx]
        otus.append(to_otu)
        con = ''
        if is_con:
            con = '; '.join(lineages[idx])
            counts = data
        else:
            counts = data
        taxonomy.append(con)
        if not normalize:
            for i,c in zip(label_list,counts):
                if i in samples_from_mapping:
                    label_dict[meta_dict[i][0][0]] += c
            for i in cat_list:
                new_line.append(str(label_dict[i]))
            cat_otu_table.append(new_line)

        else:
            new_line.extend(counts)
            norm_otu_table.append(new_line)
            for i, c in zip(label_list,counts):
                sample_counts[i] += c
    total = 0
    if normalize:
        for l in norm_otu_table:
            counts = l
            new_line = []
            label_dict = defaultdict(float)
            getcontext().prec = 28
            for i,c in zip(label_list,counts):
                if i in samples_from_mapping:
                    label_dict[meta_dict[i][0][0]] += float(c)/(sample_counts[i])
            for i in cat_list:
                new_line.append(round((label_dict[i]/ num_samples_by_cat[(category,i)]),5))
            cat_otu_table.append(new_line)
    return  cat_otu_table, otus, taxonomy
```

Panel D: QIIME 1.4.0: `Qiime/qiime/summarize_otu_by_cat.py` (continued; prior to switch to `biom-format` Table objects).

```
def summarize_by_cat(map_lines,otu_sample_lines,category,norm):
    """creates the category otu table"""
    cat_by_sample, sample_by_cat, num_meta, meta_dict, label_lists_dict, \
                   num_samples_by_cat = get_sample_cat_info(map_lines,category)

    lines, otus, taxonomy = get_counts_by_cat(otu_sample_lines, num_meta, \
                  meta_dict,label_lists_dict[category],category,num_samples_by_cat,\
                  norm)

    #This for loop was added to remove columns that sum to 0, since you may
    #pass a mapping file that has more samples than in the OTU table, hence resulting
    #in columns with no counts
    new_labels=[]
    new_lines=[]
    for i,line in enumerate(zip(*lines)):
        total_col=sum([float(x) for x in line])
        if total_col>0:
            new_lines.append(line)
            new_labels.append(label_lists_dict[category][i])
    new_lines=zip(*new_lines)

    lines = format_otu_table(new_labels, otus, array(new_lines), \
                  taxonomy=taxonomy,
                  comment='Category OTU Counts-%s'% category)
    return lines
```

Panel E: QIIME 1.4.0-dev, revision 2770: `Qiime/scripts/summarize_otu_by_cat.py` Replacement for all code in Panels A-D after switch to `biom-format` Table objects from native QIIME OTU table data structures.

```
    # define a function that returns the bin a sample shouldbe placed into
    bin_function = lambda sample_metadata: sample_metadata[mapping_category]
    # parse the sample metadata and add it to the OTU table (we assume that
    # sample metadata is not already present in the table)
    sample_metadata = parse_mapping_file_to_dict(open(mapping_fp,'U'))[0]
    table = parse_biom_table(open(otu_table_fp,'U'))
    table.addSampleMetadata(sample_metadata)
    # create a new OTU table where samples are binned based on their return
    # value from bin_function
    result = table.collapseSamplesByMetadata(bin_function,norm=False,min_group_size=1)

    # normalize the result if requested by the user
    if normalize:
        result = result.normObservationBySample()

    # write a new BIOM file
    f = open(output_fp,'w')
    f.write(format_biom_table(result))
    f.close()
```
