# Supplementary figures and images for: The Biological Observation Matrix (BIOM) format or: how I learned to stop worrying and love the ome-ome
Source: Gigascience. 2012 Jul 12;1:7. doi: 10.1186/2047-217X-1-7 (PMC3626512; doi:10.1186/2047-217X-1-7)

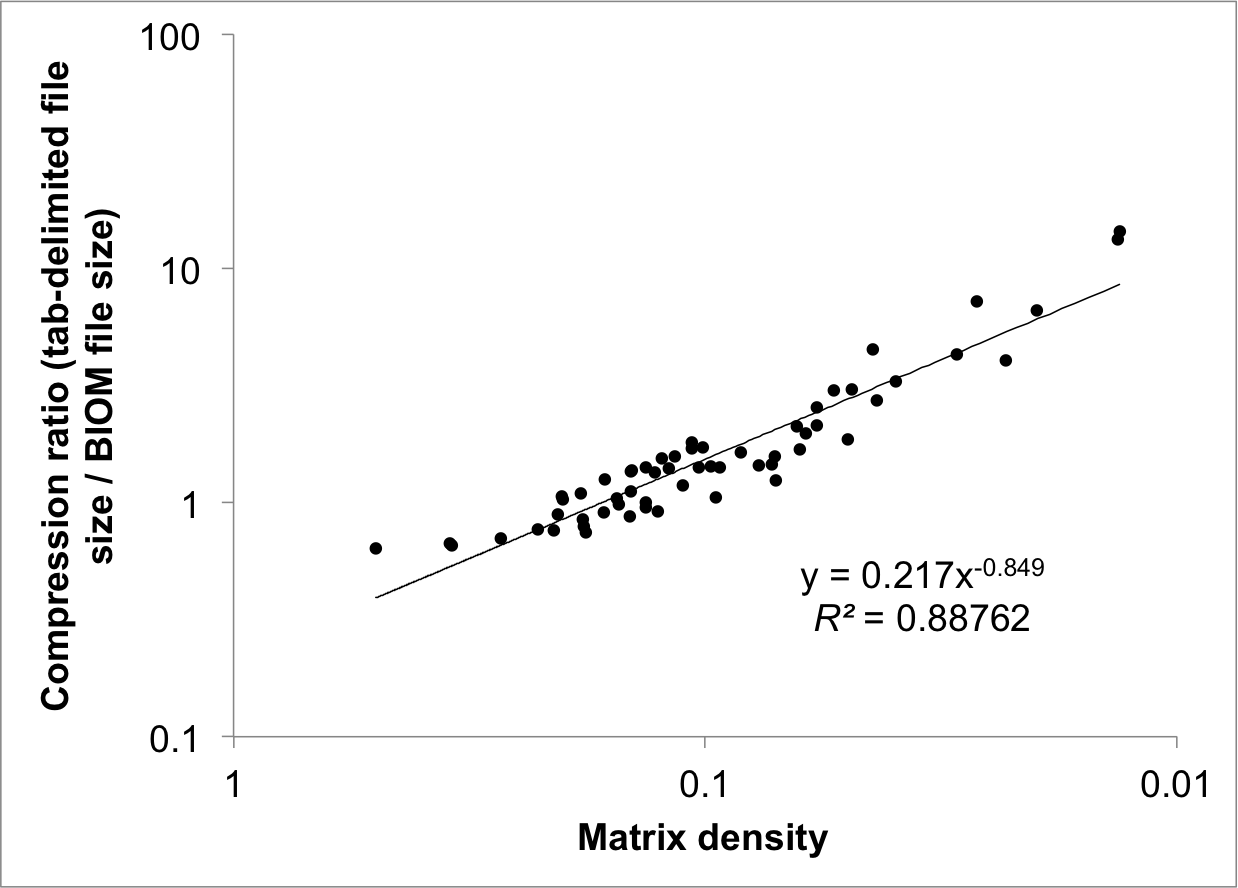

Supplement: Additional file 4: Data 1. — Representative OTU tables in BIOM and classic QIIME OTU table format. [file 2047-217X-1-7-S4.png]
