# Supplementary material for: The Biological Observation Matrix (BIOM) format or: how I learned to stop worrying and love the ome-ome
Source: Gigascience. 2012 Jul 12;1:7. doi: 10.1186/2047-217X-1-7 (PMC3626512; doi:10.1186/2047-217X-1-7)
Supplement: Additional file 6: Figure S1. — Matrix density versions compression ratio. [file 2047-217X-1-7-S6.pdf]

```
{
 "id":null,
 "format": "Biological Observation Matrix 0.9.1",
 "format_url": "http://biom-format.org",
 "type": "OTU table",
 "generated_by": "QIIME revision 1.4.0-dev",
 "date": "2011-12-19T19:00:00",
 "rows":[
    {"id":"GG_OTU_1", "metadata":
      {"taxonomy":["k__Bacteria", "p__Proteobacteria",
                   "c__Gammaproteobacteria", "o__Enterobacteriales",
                   "f__Enterobacteriaceae", "g__Escherichia", "s__"]}},
    {"id":"GG_OTU_2", "metadata":
      {"taxonomy":["k__Bacteria", "p__Cyanobacteria",
                   "c__Nostocophycideae", "o__Nostocales",
                   "f__Nostocaceae", "g__Dolichospermum", "s__"]}},
    {"id":"GG_OTU_3", "metadata":
      {"taxonomy":["k__Archaea", "p__Euryarchaeota",
                   "c__Methanomicrobia", "o__Methanosarcinales",
                   "f__Methanosarcinaceae", "g__Methanosarcina", "s__"]}},
    {"id":"GG_OTU_4", "metadata":
      {"taxonomy":["k__Bacteria", "p__Firmicutes",
                   "c__Clostridia", "o__Halanaerobiales",
                   "f__Halanaerobiaceae", "g__Halanaerobium",
                   "s__Halanaerobiumsaccharolyticum"]}},
    {"id":"GG_OTU_5", "metadata":
      {"taxonomy":["k__Bacteria", "p__Proteobacteria",
                   "c__Gammaproteobacteria", "o__Enterobacteriales",
                   "f__Enterobacteriaceae", "g__Escherichia", "s__"]}}
    ],
 "columns":[
    {"id":"Sample1", "metadata":{
                             "BarcodeSequence":"CGCTTATCGAGA",
                             "LinkerPrimerSequence":"CATGCTGCCTCCCGTAGGAGT",
                             "Description":"human gut"}},
    {"id":"Sample2", "metadata":{
                             "BarcodeSequence":"CATACCAGTAGC",
                             "LinkerPrimerSequence":"CATGCTGCCTCCCGTAGGAGT",
                             "Description":"human gut"}},
    {"id":"Sample3", "metadata":{
                             "BarcodeSequence":"CTCTCTACCTGT",
                             "LinkerPrimerSequence":"CATGCTGCCTCCCGTAGGAGT",
                             "Description":"human gut"}},
    {"id":"Sample4", "metadata":{
                             "BarcodeSequence":"CTCTCGGCCTGT",
                             "LinkerPrimerSequence":"CATGCTGCCTCCCGTAGGAGT",
                             "Description":"human skin"}},
    {"id":"Sample5", "metadata":{
                             "BarcodeSequence":"CTCTCTACCAAT",
                             "LinkerPrimerSequence":"CATGCTGCCTCCCGTAGGAGT",
                             "Description":"human skin"}}
            ],
 "matrix_type": "sparse",
 "matrix_element_type": "int",
 "shape": [5, 5],
 "data":[[0,2,1],
        [1,0,5],
        [1,1,1],
        [1,3,2],
        [1,4,3],
        [2,2,1],
        [2,3,4],
        [3,0,2],
        [3,1,1],
        [3,2,1],
        [4,1,1],
        [4,2,1]
       ]
}
```
