# Supplementary material for: The Biological Observation Matrix (BIOM) format or: how I learned to stop worrying and love the ome-ome
Source: Gigascience. 2012 Jul 12;1:7. doi: 10.1186/2047-217X-1-7 (PMC3626512; doi:10.1186/2047-217X-1-7)
Supplement: Additional file 7: Data 2. — Version 1.0.0 of the biom-format software package. [file 2047-217X-1-7-S7.tgz › biom-format-1.0.0/doc/_templates/layout.html]

{% extends "!layout.html" %}
{# include the Google Analytics Tracker #}
{% block footer %}
{{ super() }}
{% endblock %}
